# Towards Porous Silicon Oxycarbide Materials: Effects of Solvents on Microstructural Features of Poly(methylhydrosiloxane)/Divynilbenzene Aerogels

**DOI:** 10.3390/ma11122589

**Published:** 2018-12-19

**Authors:** Susana Aguirre-Medel, Prasanta Jana, Peter Kroll, Gian Domenico Sorarù

**Affiliations:** 1 Department of Chemistry and Biochemistry, The University of Texas at Arlington, 700 Planetarium Place, Arlington, TX 76019, USA; susana.aguirre-medel@mavs.uta.edu; 2 Department of Industrial Engineering, University of Trento, Via Sommarive 9, I-38123 Trento, Italy; pjanaigcar137@gmail.com

**Keywords:** supercritical drying, aerogel, preceramic polymers

## Abstract

We investigate the impact of solvents on the microstructure of poly(methylhydrosiloxane)/divinylbenzene (PMHS/DVB) aerogels. The gels are obtained in highly diluted conditions via hydrosilylation reaction of PMHS bearing Si-H groups and cross-linking it with C=C groups of DVB. Polymer aerogels are obtained after solvent exchange with liquid CO_2_ and subsequent supercritical drying. Samples are characterized using microscopy and porosimetry. Common pore-formation concepts do not provide a solid rationale for the observed data. We postulate that solubility and swelling of the cross-linked polymer in various solvents are major factors governing pore formation of these PMHS/DVB polymer aerogels.

## 1. Introduction

Aerogels are a class of porous solids first synthesized in 1931 by Kistler [1]. Aerogels refer to wet gels in which the liquid is later replaced with air resulting in a solid with little shrinkage. Application fields of aerogels include catalysis, thermal and electric insulation, water filtering and many more [2]. Although the Polymer Derived Ceramic (PDC) route has been extensively used for synthesizing Si-based ceramics it was not until recently that SiC, SiCN and SiOC aerogels were synthesized using the PDC route [3,4]. In this process, first a polymer aerogel is synthesized and then converted, through a pyrolysis process in inert atmosphere, into the corresponding PDC aerogel. The polymer aerogel is synthesized via hydrosilylation of a silicon-based polymer bearing Si-H bonds with molecules bearing C=C moieties [5]. The cross-linking is performed in highly diluted conditions.

PDC aerogels have been synthesized and characterized for gas sensing [6], anodes for Li-ion batteries [7], water purification [8], and electromagnetic adsorbers [9]. For example, SiCO aerogels were found to show good sensing response towards N_2_ at 300 °C and H_2_ at 500 °C [6]. In a separate study, SiCO aerogels showed high specific reversible capacity of more than 900 mAh/g with 360 mAh/g (10C) charging rate [7].

In this study we explore the role of the solvent in the synthesis of polymer aerogels made from poly(methylhydrosiloxane) and divinylbenzene (PMHS/DVB) aerogels. Accordingly, four different types of organic solvent (n-hexane, cyclo-hexane, tetrahydrofurane, and acetone) have been used while keeping all the other synthesis parameters fixed.

## 2. Methods

Poly(methylhydrosiloxane), PMHS (MW~1900, CAS: 63148-57-2) and divinylbenzene (DVB, technical grade, 80%, CAS: 1321-74-0) were purchased from Alfa Aesar (Alfa Aesar Ward Hilt, Haverhill, MA, USA). The solvents, cyclohexane and acetone, were bought from J. T. Baker (Fisher Scientific Italia, Rodano, Italy), tetrahydrofuran (THF) from Sigma-Aldrich (Sigma-Aldrich, Saint Louis, MO, USA) and n-hexane from Chem-Lab (Chem-Lab, West-Vlaanderen, Belgium). The catalyst (platinum divinylmethylsiloxane complex, ~2% in xylene (CAS: 68478-92-2) was bought from Sigma Aldrich, Saint Louis, MO, USA. All the chemicals were used as purchased without any further purification steps.

All samples were prepared with a 1:2 mass ratio of PMHS to DVB. In a standard reaction 0.5 g (0.47 cm^3^) PHMS and 1.0 g (0.93 cm^3^) DVB were mixed in 5.5 g of solvent. The mixture was homogenized with a magnetic stirrer for two minutes before 10 µL of the Pt catalyst were added under stirring for another two minutes. Thereafter the mixture was transferred into a pressure reactor (Parr acid digestion vessel; model 4749 Parr, Moline, IL, USA) and placed into a furnace at 150 °C for 6 h. After crosslinking, the sample was taken off the furnace and allowed to cool to room temperature. The wet gel was extracted from the container and washed five times with solvent to eliminate unreacted reagents and catalyst. Thereafter, the wet gel was transferred into a home-made CO_2_-reactor equipped with two glass windows that allow visual control of the solvent exchange with CO_2_ and supercritical drying process. Inside the reactor, the sample was washed with liquid CO_2_ at 10 °C twice a day for a total of 10 solvent exchanges. As the final step of the drying process, the temperature in the dryer was slowly increased to 45 °C at a pressure of 100–110 bar. Supercritical drying was then performed overnight. The complete procedure was performed using THF, cyclohexane, n-hexane, and acetone as solvents.

Porosity characterization was performed by N_2_ physisorption at −196 °C with a Micromeritics ASAP 2010 instrument (Micromeritics, Norcross, GA, USA). Specific surface area (SSA) of the samples was determined in the relative pressure (p/p_0_) range between 0.05 and 0.30 using the Brunauer–Emmett–Teller approach (BET). The total pore volume (TPV) was calculated as TPV = VαD, where Vα is the volume adsorbed at P/P_0_ 0.99, and D equals the density conversion factor (0.0015468) for the nitrogen gas as adsorbate gas. The assumption that the pores are open-ended and cylindrical is used. The average pore size is calculated using the equation 4000 TPV/SSA with the average pore size given in nanometers.

Microstructural characterization was performed by acquiring Field Emission Scanning Electron Microscopy, FE-SEM, images of the fracture surface of PDC aerogels with a Zeiss supra 60 equipment (Carl Zeiss NTS GmbH, Oberkochen, Germany) operating in high-vacuum mode at 2.00 kV and after sputtering the samples with a thin gold film.

## 3. Results

**Figure 1:**
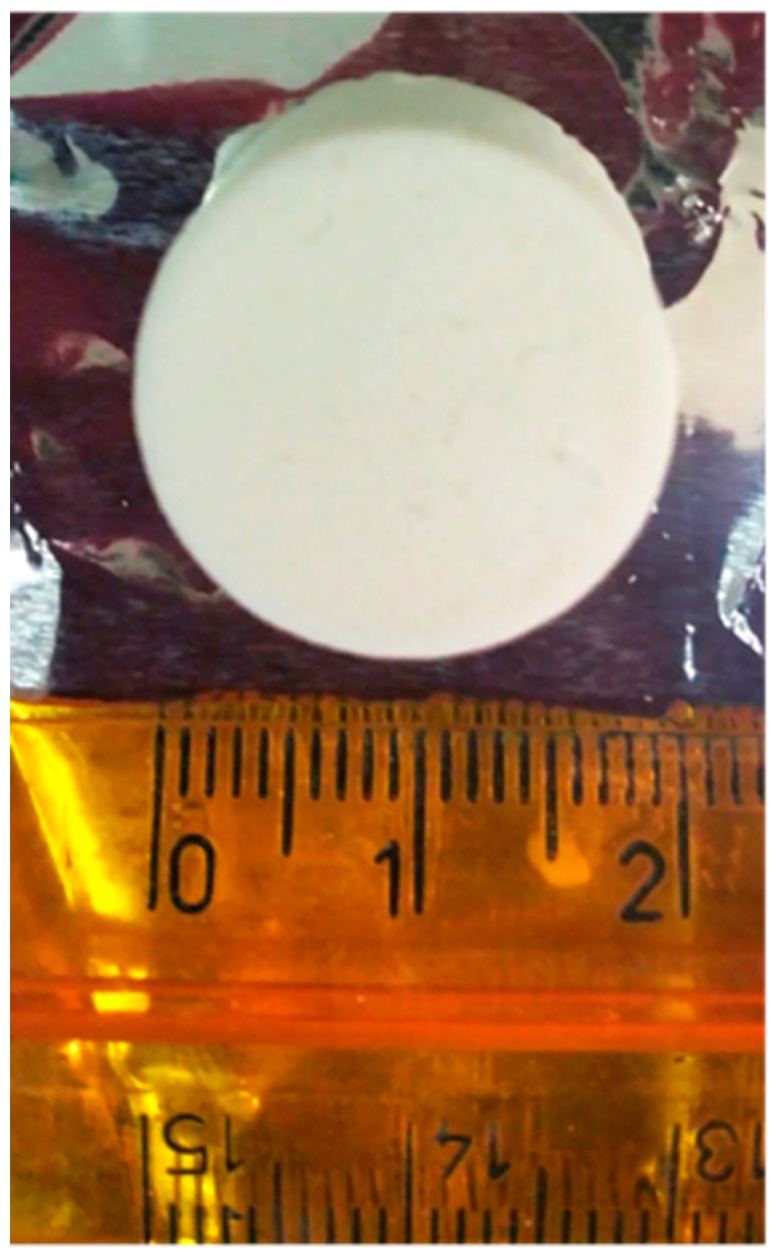
Polymeric poly(methylhydrosiloxane)/divinylbenzene (PMHS/DVB) aerogels synthesized in n-hexane after supercritical drying.

After aging in the digestion vessel the samples appear white. The appearance does not change after supercritical drying (Figure 1).

Extracting the solvent in the supercritical dryer causes significant shrinkage of samples. To account for the shrinkage, we measured height and diameter of the (almost) cylindrical samples before and after supercritical drying using a caliper. Bulk densities of samples were determined using measured volume and mass. As a caveat, these measurements carry a substantial error margin. Nevertheless, relative shrinkage and density data aligns with other quantitative data shown in Table 1 and displays two groups of polymer aerogels: samples synthesized in cyclohexane and THF exhibit linear shrinkage of over 30%, while samples synthesized in acetone and n-hexane contract only about 25%. Linear shrinkage correlates with bulk density of the aerogel. Densities of the first group (0.61–0.73 g/cm^3^) are about twice as high than those of the second (0.27–0.38 g/cm^3^). The shrinkage data agrees with previous syntheses carried out using acetone and cyclohexane as solvents [7]. Necessary for the later discussion is the observation that in our experiments the shrinkage occurs inside the CO_2_ reactor already during the first washing with liquid CO_2_. Although we cannot measure the linear dimension of the sample inside the reactor, we get the visual impression that supercritical extraction of CO_2_ in the drying process does not cause further significant shrinkage, but that all changes in size happened before.

**Figure 2:**
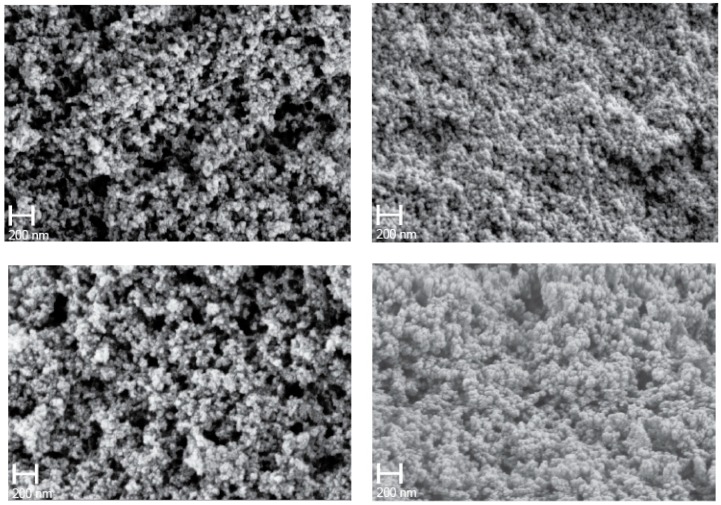
FE-SEM micrographs of PMHS/DVB polymer aerogels synthesized in cyclohexane (**top left**), tetrahydrofuran (**top right**), acetone (**bottom left**), and n-hexane (**bottom right**). All pictures were obtained using the same magnification (100 k).

Differences in microstructure of the polymer aerogels are discernable in FE-SEM images of the samples shown in Figure 2. The porous microstructure of polymer aerogels is typically explained by aggregation of small particles [10,11]. Characteristic particle diameters and pore sizes of the samples appear quite different. In particular, the sample synthesized in THF displays a fine microstructure in comparison to the coarser microstructure of the sample synthesized in n-hexane.

**Figure 3:**
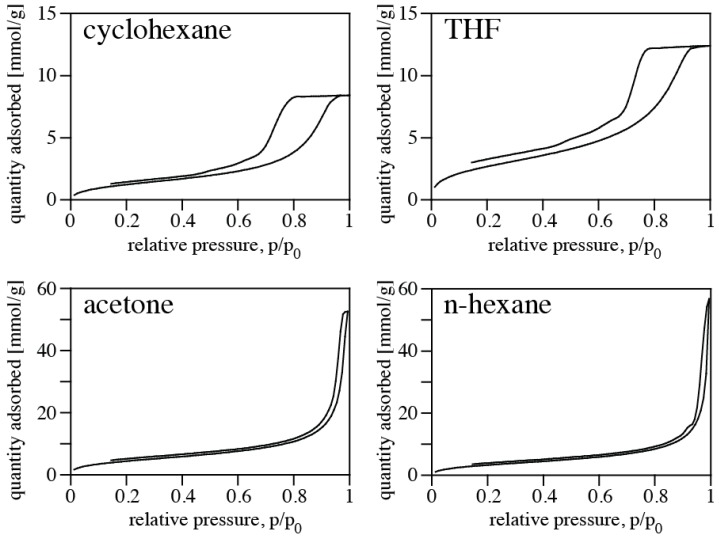
Nitrogen sorption isotherms (at 77 K) of PMHS/DVB polymer aerogels synthesized in cyclohexane (**top left**), tetrahydrofuran (**top right**), acetone (**bottom left**), and n-hexane (**bottom right**).

**Figure 4:**
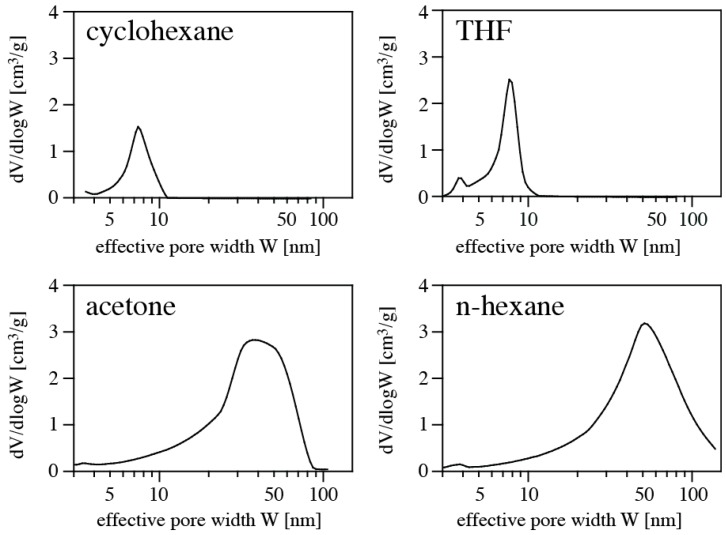
Pore size distributions of PMHS/DVB polymer aerogels synthesized in cyclohexane (**top left**), tetrahydrofuran (**top right**), acetone (**bottom left**), and n-hexane (**bottom right**).

Nitrogen isotherms of PMHS/DVB polymer aerogels synthesized in various solvents are shown in Figure 3, and pore size distributions are shown in Figure 4. Results of the porosimetry and surface characterization for the different samples are given in Table 1. We find the highest SSA of 392 m^2^/g for the aerogel synthesized in acetone, and the lowest SSA (120 m^2^/g) for using cyclohexane as solvent. The smallest average pore diameter in a sample is 4.2 nm (THF), and the largest 26.5 nm (cyclohexane). The highest pore volume of an aerogel is 1.99 cm^3^/g using n-hexane as solvent, and the lowest occurs for using THF. Qualitatively, the values and trends are consistent with apparent microstructures displayed in FE-SEM pictures (Figure 2). Samples obtained using cyclohexane or THF as solvent exhibit a denser microstructure and smaller pore sizes, consistent with the higher density. This is complemented by small pore volume, small average pore diameter, and small specific surface area. On the other hand, samples synthesized in acetone and n-hexane display a more open porous microstructure, consistent with a lower measured density. Porosimetry of these samples yield larger pore volume, larger average pore diameter, and high specific surface area. The observed porosities are typical for silica-based aerogels [12]. Pore and surface characteristics fall into the range of data reported for polymer aerogels from polysiloxane and polycarbosilane precursors [3]. In particular, our results are consistent with previous experiments of aerogels from PMHS and DVB [7].

## 4. Discussion

**Figure 5:**
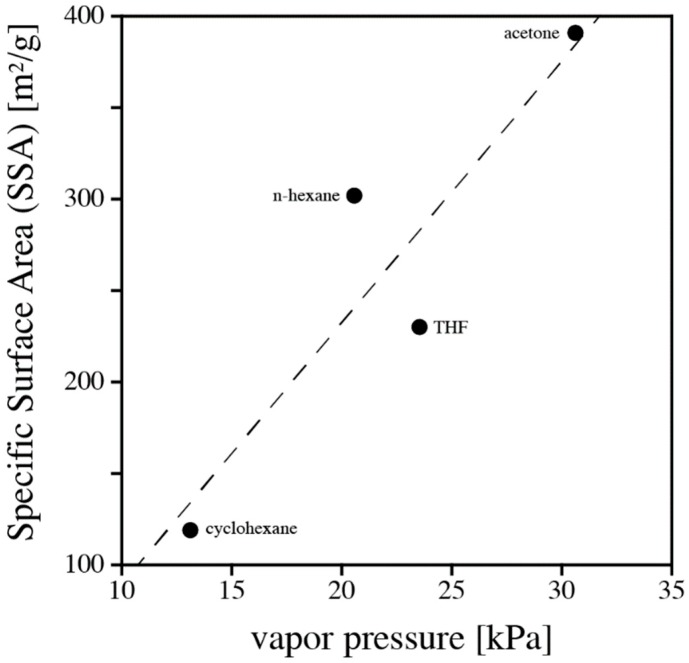
SSA plotted as a function of vapor pressure (at 25 °C) of solvent for the four solvents used in this study. The straight line is given to guide the eye.

The use of solvents to modify and control porosity of silica aerogels is well documented in the literature [13,14,15]. Solvents impact reaction rates during gel formation, capillary stresses during drying, and, ultimately, the structure of final products. Mixtures of polar and non-polar solvents have been used to modify the gelation process and tailor pore structures of silica xerogels [13]. No similar data exists in the literature for polymer aerogels. In search of a rationale for the data collected in Table 1, we related various solvents properties to the aerogel characteristics (SSA, average pore diameter, pore volume). Among those, vapor pressure of solvent correlates best with SSA of the aerogel; see Figure 5. However, vapor pressure alone does not offer a rationale to explain the observed trends.

We became more successful by considering solubility of the PMHS/DVB polymer system in the respective solvent. Solubility is intertwined with the degree of swelling of a polymer network in a solvent. We will use both concepts—solubility and swelling—to provide a rationale for porosity and surface characteristic of the PMHS/DVB polymer aerogels.

Solubility of polymers is best described using Hansen’s approach to solubility [16,17]. The three Hansen parameters, *δ_d_*, *δ_p_* and *δ_h_*, provide a semiquantitative measure of nonpolar (*δ_d_*), polar (*δ_p_*), and hydrogen-bond (*δ_h_*) interactions for a system. They span the three-dimensional Hansen Solubility Parameter (HSP) space, and any molecular compound is represented by a point in HSP space. Mutual solubility is quantified by the distance *R_A_* between two compounds (index 1 and 2) in HSP space: $R_{A}=\sqrt{4{\left(\delta_{d1}-\delta_{d2}\right)}^{2}+{\left(\delta_{p1}-\delta_{p2}\right)}^{2}+{\left(\delta_{h1}-\delta_{h2}\right)}^{2}}$. Essentially, for a specific solute a “good” solvent is found in short distance *R_A_*, while a “bad” solvent has a large *R_A_*. The set of “good” solvents for a given solute falls within a sphere, termed Hansen sphere, while solvents considered “bad” are located outside that sphere. Note that the radius of Hansen spheres of different solutes can be different. Moreover, since the parameters are related to Gibbs energies, Hansen spheres ultimately depend on temperature and pressure.

For solvents used in this study, HSPs are listed in Table 2. Unfortunately, no solubility parameters exist of the PMHS/DVB system. We estimate the parameters from small molecule data (e.g., styrene) and data for polydimethylsiloxane (PDMS). Hansen parameters of styrene are *δ_d_* = 18.6, *δ_p_* = 1.0, *δ_h_* = 4.1 [18]. Multiple parameters have been reported for PDMS, for which solubility in organic solvents depends on the degree of cross-linking (molecular weight) and temperature. For a high-weight (long chain) PDMS polymer, Hansen parameters of *δ_d_* = 17, *δ_p_* = 4, *δ_h_* = 4 have been suggested [19]. Combining data for styrene and PDMS, we find that solubility parameters of the PMHS/DVB system likely fall into the range *δ_d_* = 17–18.5, *δ_p_* = 1–5, *δ_h_* ≈ 4, with some uncertainty about the exact values. It turns out that even with this uncertainty, the distinction between THF and cyclohexane as solvents for the PMHS/DVB system on one side, and acetone and n-hexane on the other side, are well explained by their respective distance *R_A_* to PMHS/DVB in HSP space. Assuming a simple average of PDMS and styrene, we obtain solubility parameters for the PMHS/DVB polymer system of *δ_d_* = 17.8, *δ_p_* = 2.5, *δ_h_* = 4.0. Using these values, *R_A_* to cyclohexane, THF, acetone, and n-hexane, is 5.0, 5.5, 9.6 and 7.5 respectively, and a clear distinction between cyclohexane and THF on one side, and n-hexane and acetone on the other side emerges. Variations of the parameters for PMHS/DVB within the proposed range do not change the grouping. Consequently, we establish that cyclohexane and THF are better solvents for PMHS/DVB than acetone and n-hexane. For the rest of our discussion, we may simply call them “good” and “bad” solvents, respectively.

However, how does solubility of the polymer impact pore formation in the corresponding aerogel? We emphasize here the importance of swelling of a polymer for pore modification of an aerogel. Swelling of a polymer and its solubility in a solvent are intertwined. Indeed, swelling data is used to determine solubility parameters [20,21]. There is ample evidence in polymer chemistry that the higher the solubility, the higher is the degree of absorption of the solvent into the polymer and, as a consequence, the higher is the swelling of the polymer. This obviously happens for hydrogels (e.g., used in diapers). Linear swelling of a siloxane polymer (e.g., PDMS) can differ by more than a factor of two depending on the solvent [22].

**Figure 6:**
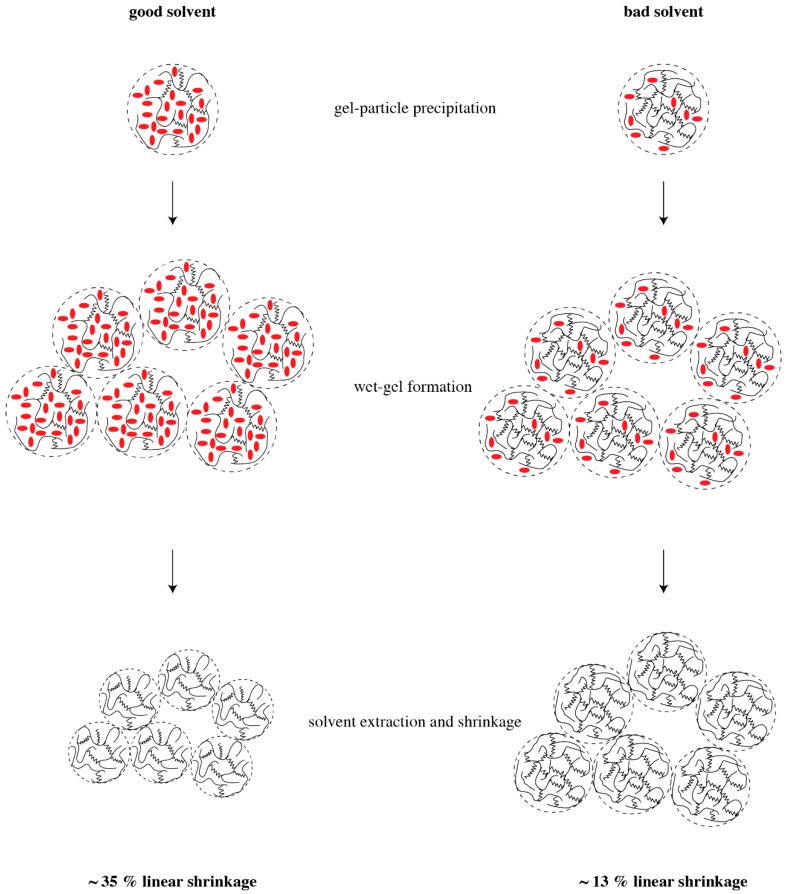
Illustration of microgel particle formation and shrinkage for PMHS/DVB polymer aerogel formation in a “good” solvent such as cyclohexane (**left**) and a “bad” solvent such as acetone (**right**). Solvent molecules are depicted in red; polymer strands are shown as black lines.

To provide a rationale for pore formation with swelled polymers, we assume that the size of precipitating microgel particles—their “radius of gyration”—depends only on the polymer itself and is independent of the solvent used. Hence, the characteristic size of the microgel particles is the same in all our experiments, no matter whether we use acetone or THF as solvent. Since a good solvent causes a high degree of swelling, the volume content of a “good” solvent—alternatively, the better swelling agent—within a gel particle is larger than that of a “bad” solvent. If the solvent is removed, the microgel particles shrink, and the higher the degree of swelling, the higher the shrinkage of the particles. The concept of microgel particle formation and shrinkage is illustrated in Figure 6. This model agrees with the data of linear shrinkage in Table 1: the higher shrinkage occurs for the “good” solvents, THF and cyclohexane, and lower shrinkage is observed for the “bad” solvents. Since the wet gel is build up by coalesced particles, the smallest particles, which originated from the better swelling agent, build up the structure with smallest average pore diameter. This conclusion agrees with the observed average pore diameters in Table 1. Since the PMHS/DVB polymer aerogels are essentially build up by similar units in the same way, and the only difference after solvent extraction is the size of the gel particles, a simple scaling yields that structures with larger shrinkage and smaller pore diameter also have the smaller SSA. This consequence should not be confused with trends according to which smaller particles yield higher SSA. SSA is a quantity specific per unit mass, and not per unit volume. According to the data in Table 1, densities of the polymer aerogels in this study differ by up to a factor of two. If indeed we are looking for the surface area per volume of sample, then we find that THF yields the highest surface area per volume of polymer aerogel.

The three solvent characteristics, namely vapor pressure, HSPs, and ability to swell a polymer are strongly correlated. Only recently, Rumens et al. [22] provided a fine study highlighting the connection between HSPs, vapor pressures of organic solvents, and the resulting degree of swelling of a polymer network based on PDMS [23]. Therefore, the correlation shown in Figure 5 only conforms to the discussion of solubility and swelling.

There may, however, be alternative paths available to explain the porosity and its relation with solubility. For instance, following standard arguments of the precipitation-polymerization mechanism, a good solvent will cause formation of larger particle before precipitation, while a bad solvent causes a small particle [24]. We would expect acetone and n-hexane to yield the smaller particle, and THF and cyclohexane to form the larger, which appears to contradict the SEM observation in Figure 2. Moreover, without fundamental differences in the particle’s internal structure, the larger nuclei will result in the larger pore size diameters, since the interstices between the nuclei are larger as for smaller nuclei as well. Smaller nuclei, produced using a “bad” solvent (acetone, n-hexane in our case), would produce smaller pore size diameters. This, however, is in opposition to the observed data in Table 2. Consequently, a precipitation-polymerization mechanism does not seem to apply in our case.

Another concept is frequently invoked for porous co-polymeric resin (PCR) materials [25,26]. As a general guide, “good” and “bad” solvent porogons separate from polymer particles at different stages of the polymerization. “Bad” solvent porogons separate early, which allows microgel particles to fuse and aggregate quickly, resulting in a coarsening of the morphology. This yields pores with large average diameter and resins with rather low SSA. “Good” solvent porogons, on the other side, separate late and microgel particles retain more of their individuality. Average pore diameters are then lower and SSA of such resins are larger. While porosity of many porous resin materials synthesized via suspension polymerization can be tailored following this reasoning [26], the explanation does not obviously apply for the PHMS/DVB system we study.

To provide further support for our hypothesis or to disprove it, we propose experiments that yield information about the size of the microgel particles during structure formation, for example static and dynamic light scattering [27,28]. A synthesis approach will be to exchange “good” and “bad” solvents during the synthesis procedure to investigate, and which step is most responsible for forming the morphology of the porous material.

## 5. Conclusions

The foregoing discussion indicates that pore formation and control of pore morphology in processing of PMHS/DVB polymer aerogels is strongly related to solubility and swelling of the polymer during synthesis. Our results show that the solvent influences porosity, average pore size, and specific surface area in a particular way, which is not explained by common pore-formation concepts. We outline a new hypothesis for pore formation of polymer aerogels synthesized by PMHS/DVB, which invokes solubility and swelling of a polymer. Further experiments, in particular with mixtures of solvents, are needed to provide additional validation to it.

## Figures and Tables

**Table 1:** Linear shrinkage (relative change of linear dimensions), density, BET specific surface area (SSA), pore volume, and average pore diameter of polymer aerogels synthesized in cyclohexane, tetrahydrofuran (THF), acetone, and n-hexane.

Sample	Relative Linear Shrinkage	Density (g/cm^3^)	SSA (m^2^/g)	Pore Volume (cm^3^/g)	Average Pore Diameter (nm)
cyclohexane	38%	0.61	120	0.59	14.7
tetrahydrofuran	31%	0.71	231	0.43	6.4
acetone	25%	0.31	392	1.82	18.8
n-hexane	24%	0.27	303	1.99	25.7

**Table 2:** Hansen parameters (units MPa^0.5^) of solvents used in this study [17]. *δ_T_* is the total solubility parameter, $\delta_{T}^{2}=\delta_{d}^{2}+\delta_{p}^{2}+\delta_{h}^{2}$.

Solvent	*δ_T_*	*δ_d_*	*δ_p_*	*δ_h_*
cyclohexane	16.8	16.8	0.0	0.2
tetrahydrofuran	19.4	16.8	5.7	8.0
acetone	19.9	15.5	10.4	7.0
n-hexane	14.9	14.9	0.0	0.0
